# Supplementary material for: Bacillus subtilis: As an Efficient Bacterial Strain for the Reclamation of Water Loaded with Textile Azo Dye, Orange II
Source: Int J Mol Sci. 2022 Sep 13;23(18):10637. doi: 10.3390/ijms231810637 (PMC9505759; doi:10.3390/ijms231810637)
Supplement: Supplementary file 1 [file ijms-23-10637-s001.zip › ijms-1886738-supplementary-done.pdf]

International Journal of
Molecular Sciences

MDPI

Supplementary Materials

Table S1. Percent degradation of orange II at different concentration.

**Concentration (ppm)**	5	10	15	20	25	30	35	40
**% Degradation**	55.67	69.28	72.31	74.85	78.96	73.11	72.28	71.50

Table S2. Orange II percent degradation at different pH.

**pH**	1	2	3	4	5	6	7	8	9	10	11	12	13	14
**% Degradation**	39.06	48.48	50.17	53.47	55.07	57.67	62.09	58.97	56.74	52.40	49.42	46.84	41.98	39.87

Table S3. Percentage degradation at different intervals of time.

**Time (Days)**	1	2	3	4	5	6	9	12	15	18	21
**% Degradation**	26.26	34.97	54.49	54.57	54.70	54.90	55	55.10	55.18	55.27	55.38

Table S4. Percentage degradation of Orange II at different range of temperature.

**Temperature(⁰C)**	25	30	35	40	45	50
**% Degradation**	33.14	52.94	63.34	50.14	46.20	43.11

Table S5. Percentage degradation of Orange II dye at glucose concentration.

**Concentration(mg/L)**	333.33	666.66	1000	1333.33	1666.67
**% Degradation**	60.57	68.68	71.66	70.83	70.01

Table S6. Percentage degradation of dye (Orange II) at different concentration of urea.

**Concentration(mg/L)**	333.33	666.66	1000	1333.33	1666.67
**% Degradation**	66.72	67.17	68.48	68.29	65.79

Table S7. Percent degradation of Orange II at different concentration of sodium chloride.

**Concentration(mg/L)**	333.33	666.66	1000	1333.33	1666.67
**% Degradation**	50.48	58.69	39.77	37.68	36.23

Table S8. Percent degradation of Orange II by *B.subtilis* using different Redox mediators.

**Concentration (66 mg/L)**	**Uric Acid**	**Sodium Benzoate**	**Hydroquinone**	**EDTA**
**% Degradation**	28.53	59.55	60.93	58.58
